# Dengue with Normal Platelet Count and no Hemoconcentration: Automated Hematogram in Cases with Underlying Thalassemia

**DOI:** 10.30699/IJP.14.2.186

**Published:** 2019-06-10

**Authors:** Beuy Joob, Viroj Wiwanitkit

**Affiliations:** 1 *Ph.D., Sanitation 1 Medical Academic Center, Bangkok, Thailand*; 2 *Honorary Professor, Dr DY Patil University, Pune, India*

**Dear Editor,**

Dengue is an important arbovirus infection. This infection can result in an acute febrile illness. The important hematological abnormalities included hemoconcentration and thrombocytopenia (1). Due to the decreased platelet count, the patient might develop petechiae and hemorrhagic complication. In endemic area, the presumptive diagnosis of dengue is usually derived by the clinical findings (1). Sometimes, the atypical clinical presentation of dengue can be seen. The dengue without thrombocytopenia is possible and might be difficult for diagnosis (2).

Here, the authors present an interesting case of dengue with platelet count and no hemocon-centration. The automated hematogram can help explain the aberrant complete blood count finding. The patient was a 13 years old female patient. The chief complaint was high fever for 4 days and petechiae for 1 day. The tourniquet test was positive. The complete blood count was done and the hemoglobin level was 12.4 g/dL and platelet count was 276,000/mm^3^. In the present case, there was no thrombocytopenia and no hemo-concentration. However, the autoamted hematogram (Figure 1) showed flag that platelet interpretation was possible. From history taking, the patient was a known case of beta-thalassemia/hemoglobin E disorder. The additional dengue NS1 Ag test was positive. The patient was diagnosed to have dengue and received the standard fluid replacement therapy. She got full recovery within 1 week.

In the present case, the unexpected normal platelet count despite overt petechiae might be explainable by the automated hematogram. The patient had the underlying hemoglobin disorder problem that results in anisopoikilocytosis and microcytic anemia. With the underlying abnormal hematological parameter, anemia, no hemoconcentration can be explained. Regarding the platelet count, the microcytosis, anisocytosis and poikilocytosis can interfere with the platelet count in autoamted hematology analytical process. Nevertheless, the automated hematogram and flag can help explain and assist the physician in charge for further use of definitive diagnosis test for dengue.

**Figure 1:**
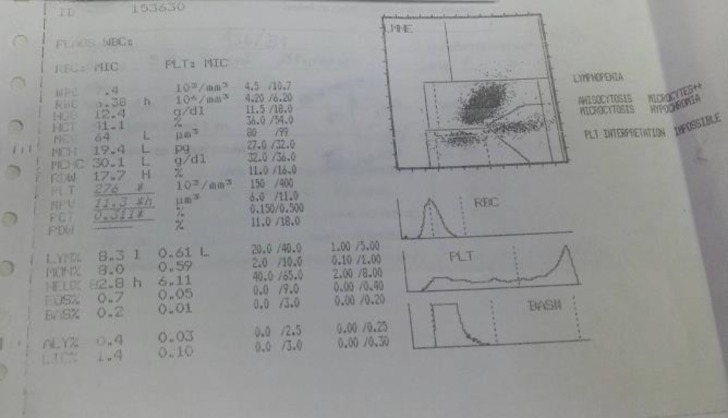
Hematogram of the patient blood test

## References

[1] Wiwanitkit V (2010). Dengue fever: diagnosis and treatment. Expert Rev Anti Infect Ther.

[2] Joob B, Wiwanitkit V (2016). Normal platelet count is common among early dengue patients confirmed by the nonstructural protein 1 antigen test. Ann Trop Med Public Health.

